# Anatomical variation of co-existence of 4^th ^and 5^th ^short metacarpal bones, sesamoid ossicles and exostoses of ulna and radius in the same hand: a case report

**DOI:** 10.1186/1757-1626-1-281

**Published:** 2008-10-29

**Authors:** Alexandros Tzaveas, Georgios Paraskevas, Christos Gekas, Aristeidis Vrettakos, Konstantinos Antoniou, Ioannis Spyridakis

**Affiliations:** 1 Orthopaedic Department, "Panagia" Hospital, Nik. Plastira 22, N. Krini, 55132, Kalamaria, Thessaloniki, Greece; 2 Department of Anatomy, Medical Faculty, Aristotle University of Thessaloniki, P.O. Box 300, 54124, Thessaloniki, Greece

## Abstract

**Introduction:**

The anatomical variations of bones in the hand are common. The existence of exostosis and shortening of metacarpal bones has been described in the literature as part of the hereditary multiple exostosis syndrome but no case has been reported with the co-existence of sesamoid ossicles in the same patient.

**Case presentation:**

We report a case with co-existence of distal ulnar and radial exostoses, 4^th ^and 5^th ^short metacarpals and sesamoid ossicles in the wrist area.

**Conclusion:**

This variation may help the interpretation of pain or sensory disorders in the hand and wrist areas.

## Introduction

The anatomical variations of bones in the hand are usually related to the presence of pain caused by pressure over the tendons or the joint capsules. Occasionally sensory disorders involving the median nerve or the ulnar nerve may be present. Usually these kind of symptoms are caused by post-traumatic bone deformities but sometimes the existence of sesamoid ossicles or exostoses may be the cause of symptoms. The frequency of sesamoid ossicles in human hand is around 0.4% – 1.6%, according to O'Rahilly's research [1]. The existence of short metacarpal bone is usually found in pseudohypoparathyroidism, in Turner syndrome and in cases with familial short stature [2-4]. The exostoses is present in patients with osteochondromatosis or multiple hereditary exostoses, which is an autosomal dominant disorder consisting of multiple cartilaginous – capped exostoses (osteochondromas) arising from the metaphyses of bones and formed by cartilage [5]. We present the co-existence of short metacarpal bones, exostoses and sesamoid ossicles in the same patient's hand, which is a rare anatomical variation. Such anatomical variations can be important for the interpretation of pain and sensory loss in some patients without history of fracture, other trauma or surgical intervention in the hand and wrist area.

## Case presentation

**Figure 1:**
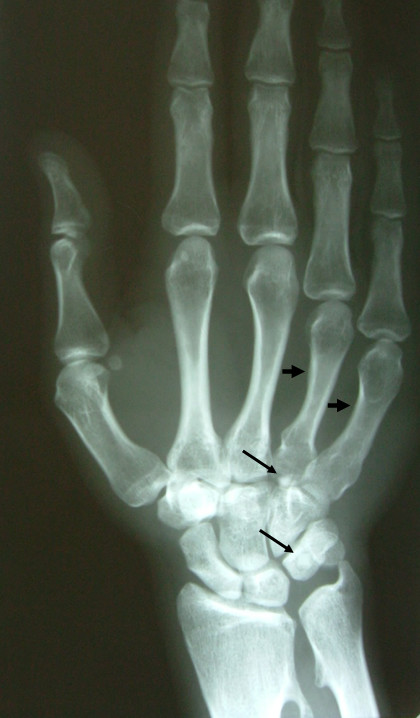
AP radiograph of the hand showing the presence of short 4^th ^and 5^th ^metacarpals (short arrows) and the presence of two sesamoids anterior to the triquetrum and hamate (long arrows).

A 44-year-old male, manual worker, non – smoker, presented to our outpatient orthopaedic department for chronic pain in the volar side of the right wrist. He was of Greek origin. His weight was 82 kg and height 1, 78 m. He had no past medical history and was social alcohol drinker. He did not reported any major family history. There was no history of trauma and no previous operation in the upper limb. Physical examination revealed tenderness on palpation of the area over the triquetrum. There was no sensory deficit involving the median nerve and no other pathological signs. Antero – posterior and lateral x-rays were performed and the co-existence of ulnar and radial exostoses together with 4^th ^and 5^th ^short metacarpal bones and two sesamoid ossicles in the wrist area were found (Fig. 1). X-ray examination of the left hand was normal. Hence, the symptoms were attributed to the presence of the one sesamoid ossicle over the triquetral bone at the volar aspect of the wrist. Pain alleviate medications were prescribed and the patient was advised to avoid heavy manual work.

**Figure 2:**
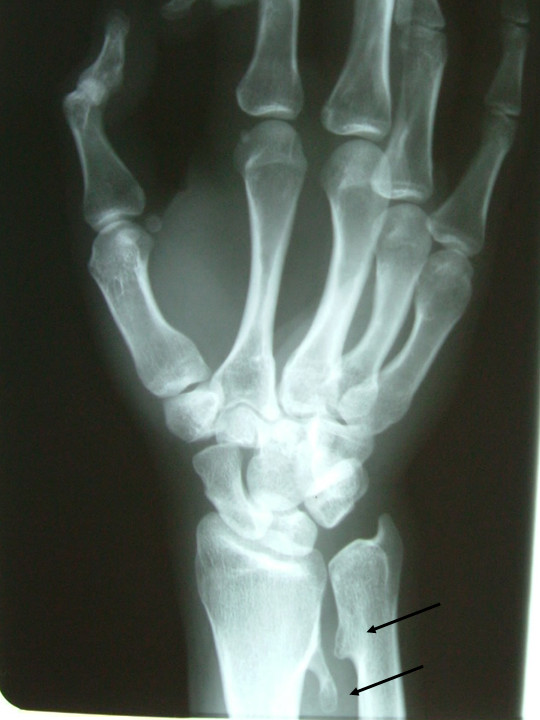
Oblique radiograph of the wrist showing the presence of two exostoses at the distal metaphysis of ulna and radius (arrows).

The exostoses arose from the distal metaphysis of the radius and ulna (Fig. 2). The radial one emerged from the ulnar side of the distal metaphysis and had a sideway and proximal direction. The length was measured to be around 2.3 cm. The ulnar exostosis emerged from the radial side, had a triangular contour and a size of about 0.5 cm.

**Figure 3:**
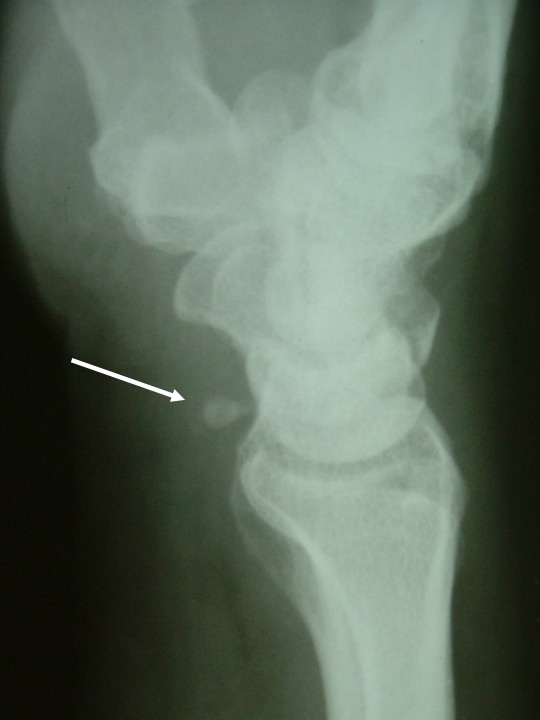
Lateral radiograph of the wrist showing the sesamoid anterior to the triquetrum.

The sesamoids were 2, both in the volar aspect of the wrist (Fig. 1 and 3). The first one was located anterior to the triquetral bone; its diameter was measured to be 0.55 cm and considered to be the cause of pain. The second one was located anterior to the hamate bone and the base of the 4^th ^metacarpal bone and its diameter was measured to be 0.48 cm. There was no pain on palpation in this area.

## Discussion

The exostoses or osteochondromas are the most frequent benign bone tumors. They constitute 20% – 50% of all benign bone tumors and 10% – 15% of all bone tumors [6,7]. They are cartilage – capped excrescences of bone that develop during physeal growth [6]. Usually they do not cause any symptoms and they constitute incidental findings. Sometimes they may cause pain or sensory disorders because of fracture, pressure upon tendons, joint capsules, nerves or vessels. These complications seem to occur more often in an autosomal dominant syndrome called "hereditary multiple exostosis" [7]. This syndrome appears with multiple exostoses in the metaphysis of long bones and affects individuals from early childhood until puberty. Individuals with hereditary multiple exostosis are often short in stature with varying degrees of orthopaedic deformity [8]. Malignant transformation is seen in 1% of solitary osteochondromas and in 3% – 5% of patients with hereditary multiple exostoses [7]. When malignant transformation or other complications appear, surgery may be required [8].

The incidence of the sesamoid bones in human hand is around 0.4 – 1.6% [1]. The sesamoids may be involved in fractures, luxation, subluxation, osteoarthritis, osteochondritis, tendon ruptures and pressure phenomena [9]. The sesamoid ossicle that lies under the triquetral bone is called "os hypotriquetrum" and its incidence is around 0.5% [1]. The second sesamoid bone of our case is called "os Vasalius", after the name of Vasalius, who was the first to describe a sesamoid ossicle in 1593. This particular sesamoid was primarily described by Dwight in 1907 and its frequency is around 0.3% [10].

The short metacarpals are part of many syndromes. The presence of short 4^th ^metacarpal is known as the "4^th ^metacarpal bone sign" in patients with pseudohypoparathyroidism. A short 5^th ^metacarpal may also be present in this condition [11]. Other conditions associated with short 4^th ^or 5^th ^metacarpals include Turner syndrome, hereditary multiple exostosis [11], and other less common syndromes [12]. They also appear in patients with homocystinuria [13]. Shortening of 4^th ^and 5^th ^metacarpal do not seem to be pathognomic findings in the above syndromes.

## Conclusion

Awareness of the anatomical variations of the hand may be important for the interpretation of pain and sensory deficits in this area. The presence of exostosis is something common as this kind of benign tumor is the most frequent, but they may also be part of hereditary multiple exostosis syndrome. The incidence of sesamoids in hand and wrist area is 0.4% to 1.6% [1] and they may involve many pathological conditions [9]. Shortening of the 4^th ^and 5^th ^metacarpals is also a condition that may emerge the suspicion of some syndromes like pseudohypoparathyroidism, Turner syndrome, hereditary multiple exostosis and others [11]. The co-existence of these conditions must be considered as something very rare in the literature.

## Consent

An informed written consent has been received for publication of the present case report from the patient.

## Competing interests

The authors declare that they have no competing interests.

## Authors' contributions

AT and GP examined the patient for the first time and on his follow ups. CG and KA were involved in reviewing the literature. AV and IS were involved in the research of the importance of our finding and the interpretation of the finding. AT and GP were responsible for final proof reading of the article. All authors read and approved the final manuscript.
